# Significance of Crushed Fragments and Eosinophils in Cytological Diagnosis of Hashimoto’s Thyroiditis

**DOI:** 10.7759/cureus.5851

**Published:** 2019-10-07

**Authors:** Arti Khatri, Rashmi Arora, Sumanashree Mallappa

**Affiliations:** 1 Pathology, Chacha Nehru Bal Chikitsalaya, Delhi, IND; 2 Pathology, Vardhman Mahavir Medical College and Safdarjung Hospital, Delhi, IND

**Keywords:** hashimoto thyroiditis, crushed fragments, eosinophils, morphology, cytology

## Abstract

Introduction

Hashimoto's thyroiditis (HT) is a chronic autoimmune inflammatory disorder of the thyroid gland with a prevalence of 1%-4%. The incidence of HT seems to be increasing in recent times. If serological parameters are used as sole criteria for diagnosis, cases of HT get missed or over-diagnosed. There are established cytological features of HT but that could, at times, be seen in other thyroid disorders, making cytological diagnosis difficult. The present study was undertaken to find out the significance of crushed fragments and eosinophils along with the well-known cytological features in the diagnosis of HT.

Methods

This study was carried out over a period of two years and a total of 114 patients were registered for the study; 48 patients were HT cases. The control group comprised of 66 non-thyroiditis patients. Fine-needle aspiration cytology (FNAC) was performed on all patients with palpable thyroid swelling to compare cytological features of thyroiditis (study group) with aspirates of non-thyroiditis lesions (controls).

Results

The background lymphocytes were found to be present in all cases of the study group but in variable numbers. The lymphocytes infiltrating the follicular epithelial cells were seen in most (93.75%) of the study cases. The presence of Hurthle cells was significantly higher (83%) in the study group as compared to the control group (4.5%). The presence of crushed cells morphology (crushed fragments) were seen in 40 (83.33%) of these 48 HT cases while none in the control group showed this feature. The presence of eosinophilic infiltration shows a statistically significant association with FNA diagnosis of HT patients (P<0.05).

Conclusion

The crushed fragments, if visible at low power, gives a diagnostic clue for looking up for other features of HT. Also, the crushed fragments and eosinophils could avoid the false negative and misdiagnosis of neoplasm in paucicellular and highly cellular smear respectively.

## Introduction

Thyroid diseases are one of the commonest endocrine disorders [1]. Hashimoto's thyroiditis (HT) is the most common autoimmune thyroid disorder and it is a common cause of hypothyroidism among Asians. The prevalence of HT is 1%-4% with an incidence of 30-60/1lakh population per year [2]. The incidence of HT increased 10 times over the past three decades [3]. HT is also known as chronic lymphocytic thyroiditis or autoimmune thyroiditis [2]. It commonly occurs in females with a male to female ratio of 1:5-1:7 and peak incidence is in the middle age (30-50 years) [3].HT can result in hypothyroidism and when hypothyroidism occurs in pregnancy there is an increased risk of adverse fetal outcomes [4]. Also, patients of HT are at increased risk for thyroid carcinomas and malignant lymphomas. So, it becomes essential to diagnose HT early as adequate treatment can be provided to patients. The incidence of HT detected by fine-needle aspiration (FNA) is considerably higher than when diagnosed only by serological tests [5]. Antithyroglobulin and/or anti microsomal antibodies are positive only in 60%-80% of cases of HT and 10%-15% of patients with positive antibodies may not have thyroiditis [2]. So, if serological parameters are used as sole criteria for diagnosis, cases of HT get missed or over-diagnosed. The well-known cytological markers for the diagnosis of HT include Hürthle cells, a moderate number of lymphocytes and plasma cells with scanty or no colloid but these features could be present in a variable number in other thyroid pathologies [2]. Many a time, the presence or absence of one of these features cannot confirm or negate the diagnosis of HT. The diagnosis of HT can be given based on cytological features in a clinically suspected case even if serological findings are negative. So, there is a need for additional cytological clues which will increase the sensitivity of cytological diagnosis of HT.

## Materials and methods

This study was conducted over two years on patients with palpable thyroid swelling attending the outpatient pathology department of tertiary care hospital in New Delhi, India. Ethical clearance was obtained from the Institute’s Ethical Committee. The study was a prospective observational study and included 48 study cases (HT) and 66 controls (benign Bethesda category II other than HT). Written and informed consent was taken from all the patients. Patient's identification, clinical features, and investigations including blood absolute eosinophil count (AEC) were recorded as per proforma. The patients with increased AEC of more than 350/mm3 excluded from the study. A detailed clinical history was taken which included features suggestive of hypothyroidism or hyperthyroidism, duration of thyroid swelling, and history of any sudden increase in thyroid swelling. Relevant investigations were noted which were available from the patient including thyroid-stimulating hormone (TSH) and anti-thyroid peroxidase (anti-TPO) titers.

Fine-needle aspiration cytology (FNAC) was performed on all patients with palpable thyroid swelling to compare cytological features of HT (study group) with aspirates of other benign thyroid swellings (control group). Wet ethanol-fixed smears and air-dried methanol fixed smears were stained with Papanicolaou stain and Giemsa stain respectively. The smears were studied in detail and various cytological parameters were analyzed. The patients were diagnosed as HT based on established cytological features which include: lymphocytes and plasma cells infiltrating the thyroid follicles, Hürthle cell change, increased number of lymphocytes in the background with or without lymphoid follicles [6]. Further cytomorphological features crushed fragments and eosinophils were looked in these already diagnosed cases and compared with aspirates of Bethesda category II other than HT (control group). Statistical analysis was done using SPSS 15.0 software (SPSS Inc., Chicago, IL). Chi-square test and Fischer exact test were used for categorical data. Student T-test was used for continuous variables. Univariate logistic regression was done to find out the significance of each variable. P-value < 0.05 was taken as significant.

## Results

This study was a prospective observational study done on 114 patients with palpable thyroid swelling coming to the outpatient department. The patients were categorized into two groups: 44 cases (HT) and 66 controls (benign Bethesda category II other than HT) based on the cytomorphological features. The smears of HT were studied in detail and various cytological parameters were analyzed and compared with aspirates of Bethesda category II other than HT (control). 

Out of a total of 114 study samples, about 50% of them were between the third and fifth decade of life. The mean age in our study for cases and controls are comparable and there was no significant difference between the two. Out of the 48 cases in the study group, 4% (2) were males and the rest were females (46), similarly, in the control group, 11% (7) of the patients were males and rest were females (59). A male: female ratio of 1:24 in the study group was observed. In the study group, diffuse thyromegaly was seen in 83.4% and nodular goiter in 16.6%.

The median TSH and anti-TPO titers of both the study and control group are depicted in Table 1. The serum TSH (normal range, 0.4-6.2 μU/L) levels were ranged from 0.01 to 236.8 μU/L among HT patients and from 0.01 to 113.20 μU/L in the control group. Out of the 48 HT study samples, serum anti-TPO titers (normal, up to 27I U/ml) were raised in 40 (83.33%) and among 66 patients of the control group, it was raised in 8 (12.1%).

**Table 1:** Median serum TSH and anti-TPO levels TSH: thyroid-stimulating hormone; anti-TPO: anti-thyroid peroxidase.

		Study Group	Control group	p-value
Thyroid Stimulating Hormone (TSH) (μU/L)	Median	14.00	1.50	0.001
	Range	0.01-236.80	0.01-113.20	
Anti-thyroid peroxidase (Anti-TPO) (IU/ml)	Median	134.80	22.80	0.00014
	Range	20.40-869.50	9.50-180.30	

The various cytological features of HT were compared with another benign thyroid swelling group (Table 2).

**Table 2:** Comparative analysis of various cytomorphologic features of Hashimoto’s thyroiditis with category II of the Bethesda system

Cytomorphological features	Study group		Control group		p-value	Odds Ratio (univariate)
	Freq.	%	Freq.	%		
Background Lymphocytes	48	100	7	10.61	<0.05	-
Hurthle cells	40	83.33	3	4.55	<0.05	105
Follicular invasion by Lymphocytes	45	93.75	0	0	<0.05	-
Plasma cells	19	39.58	2	3.03	<0.05	20.966
Epithelioid cells	17	35.42	8	12.12	0.001	3.976
Giant cells	12	25.00	0	0	0.00001	-
Crush Fragments	40	83.33	0	0	<0.05	-
Eosinophils	11	22.92	0	0	0.00002	-
Polymorphonuclear (PMN)	5	10.42	2	3.03	0.052	3.721
Others - Colloid	19	39.58	64	96.97	0.000	0.020
Fire Flare	1	2.08	6	9.09	0.062	0.213
Macrophages	12	25.00	30	45.45	0.013	0.400

In this study, the majority of cases of HT had smears showing moderate cellularity and hemorrhagic background with a fair number of cases showed colloid in the background (39.58%). Many of the smears in the control group also showed colloid in the background (96.9%). The background lymphocytes were found to be present in all HT cases with variable density in contrast to the control group revealing background lymphocytes in only 10.61% (p<0.05). The crushed fragments showed a significant difference among study and control group. The presence of eosinophils shows a statistically significant association with FNA diagnosis of HT patients (p<0.05).

## Discussion

HT is an autoimmune chronic inflammatory disorder of the thyroid gland. This thyroid gland is infiltrated by T and B lymphocytes which are reactive to thyroid antigens. The thyroid autoantibodies are secreted by activated B cells. The thyroid parenchyma in HT is majorly destroyed by cytotoxic T lymphocytes. In the long-standing cases of HT, the fibrosis usually replaces the follicular architecture of the thyroid gland. The active phase of the disease shows clinical features of thyrotoxicosis and usually, it is a transient phase. The evolution and destructive phases are characterized by subclinical and overt hypothyroidism [6].

The ages of the patients ranged from 8 to 70 years with the majority of patients presenting between 20-50 years with a mean age of 36 years, which is in concordance with those reported by Poropatich [7] et al. and Bhatia [6] et al. The mean age in this study for study cases and controls were comparable and there was no significant difference between the two groups.

In the present study, the majority of the patients were females in both study and control groups. A female: male ratio of 24:1 in the study group was observed. Many studies have documented a similar female predilection of HT with a female to male ratio ranging from 4:1 to 25:1 [5,7-8].

**Figure 1:**
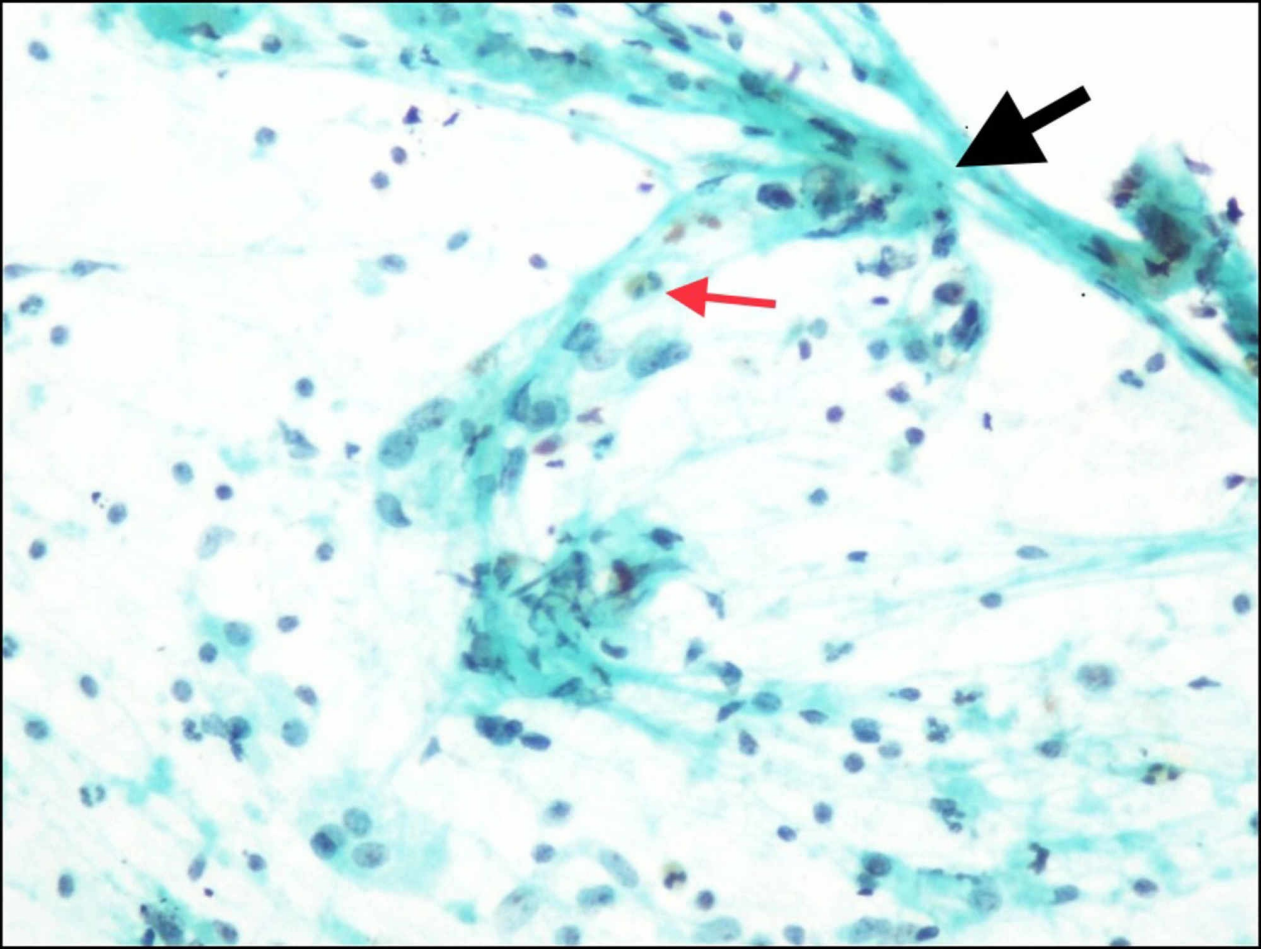
Cytology Image Hurthle cell along with the presence of lymphocytes and eosinophils (thin red arrow) and crushed fragments (thick black arrow); (Papanicolaou stain, x400).

**Figure 2:**
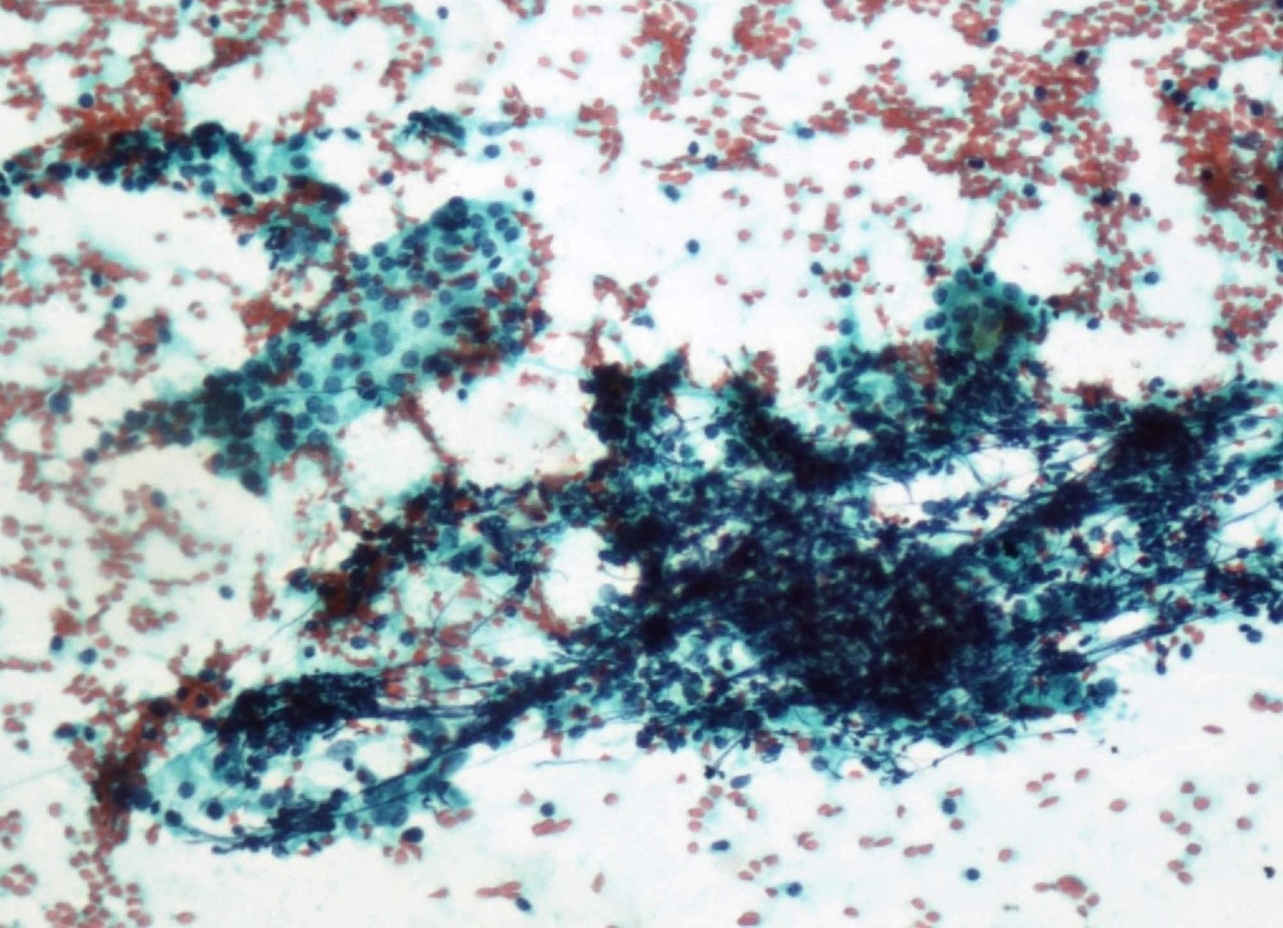
Cytology image Crushed fragments with follicular epithelial cells and lymphocytes in the background (Papanicolaou stain, 100x).

The presence of lymphocytes in HT has been reported in various studies in the past. Many authors have described the presence of lymphocytes in the smears but in variable numbers. Poropatiach [7] et al. in 1993 noticed in their study that 100% of their cases showed the presence of lymphocytes. Jayaram et al. [9] in 2007 documented in their study that about 67% of cases of HT showed the presence of lymphoid follicles. Gandhi et al. [5] noticed about 98.38% of HT cases showed the presence of lymphocytes in the smear background. In the present study, background lymphocytes were found to be present in all cases but differed in density (Figure 1). Among the control group, 10.61% of the smears also showed the presence of background lymphocytes. The presence of lymphocytes in the background can lead to over-diagnosis and needs cautious interpretation along with other cytomorphological features to avoid a false-positive diagnosis of HT. Friedman et al. [10] in 1981 noticed in their study 98% of HT showed Hürthle cell change. Poropatich et al. [7] and Pandit et al. [8] noticed Hürthle cells in 48% of their cases. Jayaram et al. [9] and Gandhi et al. [5] observed Hürthle cells in 56% and 53% of the HT cases respectively. Hürthle cells were seen in about 83% of our study group and 4.5% patients of the control group with a p-value of <0.05% (Figure 2).

Follicular cell invasion by lymphocytes is a well-established feature of HT and was reported by various studies done in the past. Jayaram et al. [9] noticed that about 69% of HT cases showed follicular cell infiltration by lymphocytes in their study done in 2007. Gandhi et al. [5] documented in their study that 93.38% of their HT cases showed follicular cell invasion by lymphocytes. In our study, 93.75% of HT cases showed follicular cell invasion by lymphocytes while none in the control group showed this feature (Figure 2).

**Figure 3:**
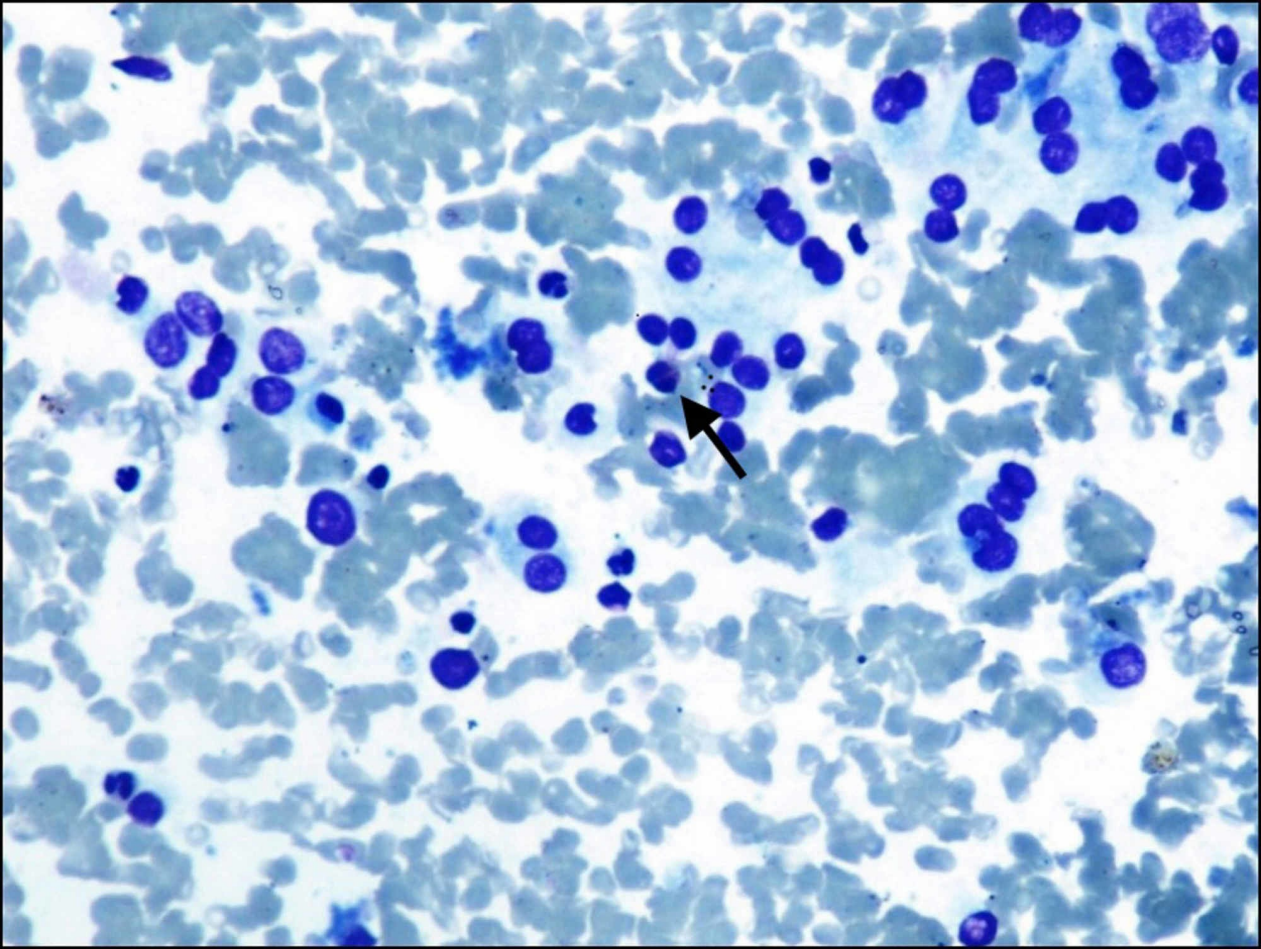
Cytology image Follicular epithelial cells with Hurthle cell change and eosinophils (arrow); (Giemsa stain, x400).

**Figure 4:**
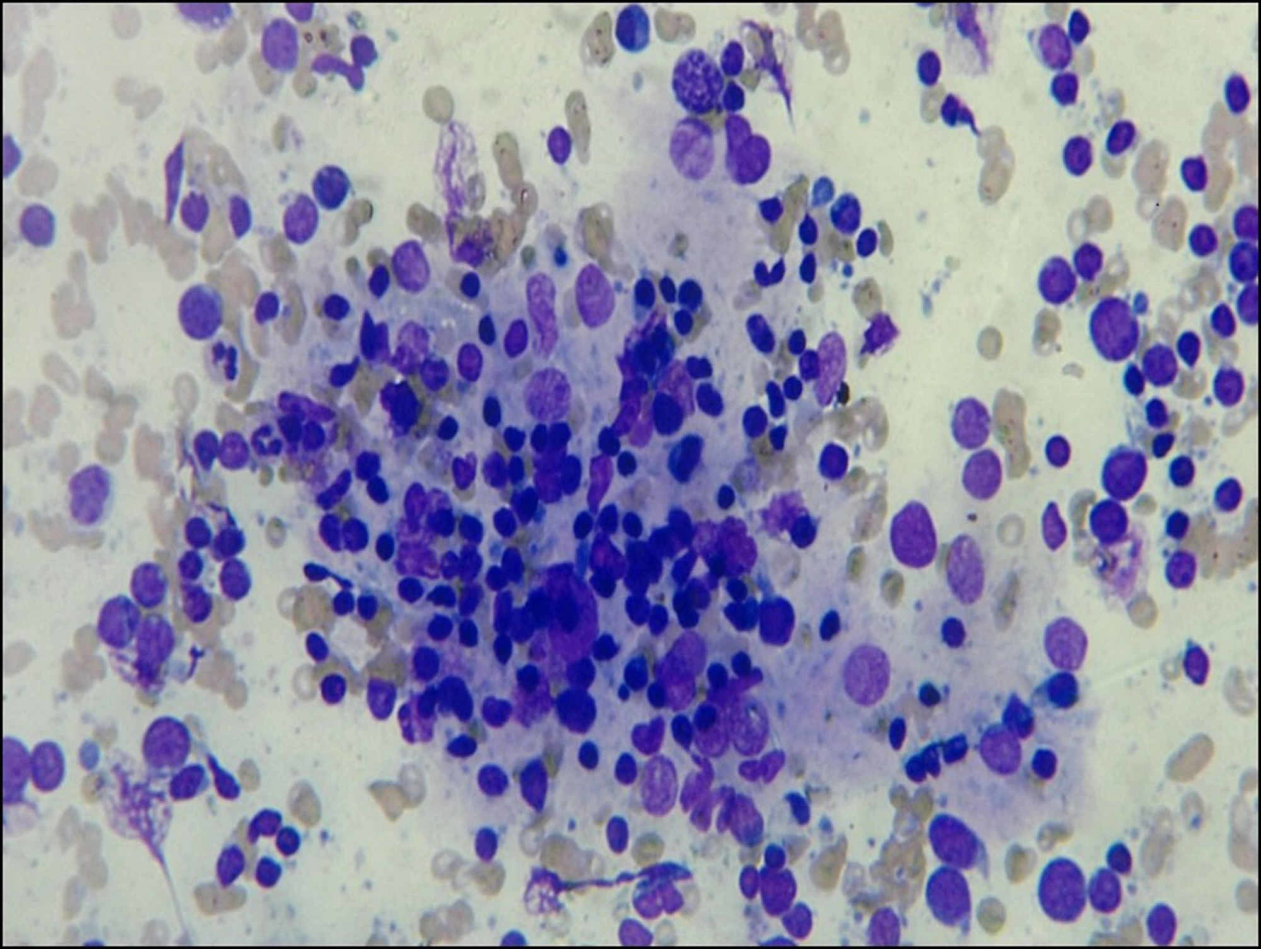
Cytology image Invasion of follicular epithelial cells by lymphocyte, Hurthle cell change also seen; (Giemsa stain, x400).

Eosinophils and polymorphs may be present in small numbers especially in the early stages of HT where the eosinophils are seen around the follicular epithelial cells [2]. The presence of eosinophils in the aspirates of HT can be explained by the presence of circulating autoantibodies and lymphocyte-derived chemotactic factors [2]. Jayaram et al. [9] in 2007 observed in their study that 17% of cases of HT showed infiltration of follicular cells by eosinophils and neutrophils. Ekambaram M et al. [2] in their study documented significant infiltration of the thyroid gland with eosinophils in cases of HT than in colloid goiter. Also, this eosinophilic infiltration was seen within the lymphoid aggregate rather in the background of smear thus it ruled out admixture with peripheral blood [2].In this study, the presence of eosinophils was seen more (23%) in the HT patients but was mainly seen in the background of the smear and not much within the follicular cell clusters (Figures 3-4). The polymorphs were observed in 10% and 3% of the HT and control group smears respectively, but, was not found to be statistically significant.

**Figure 5:**
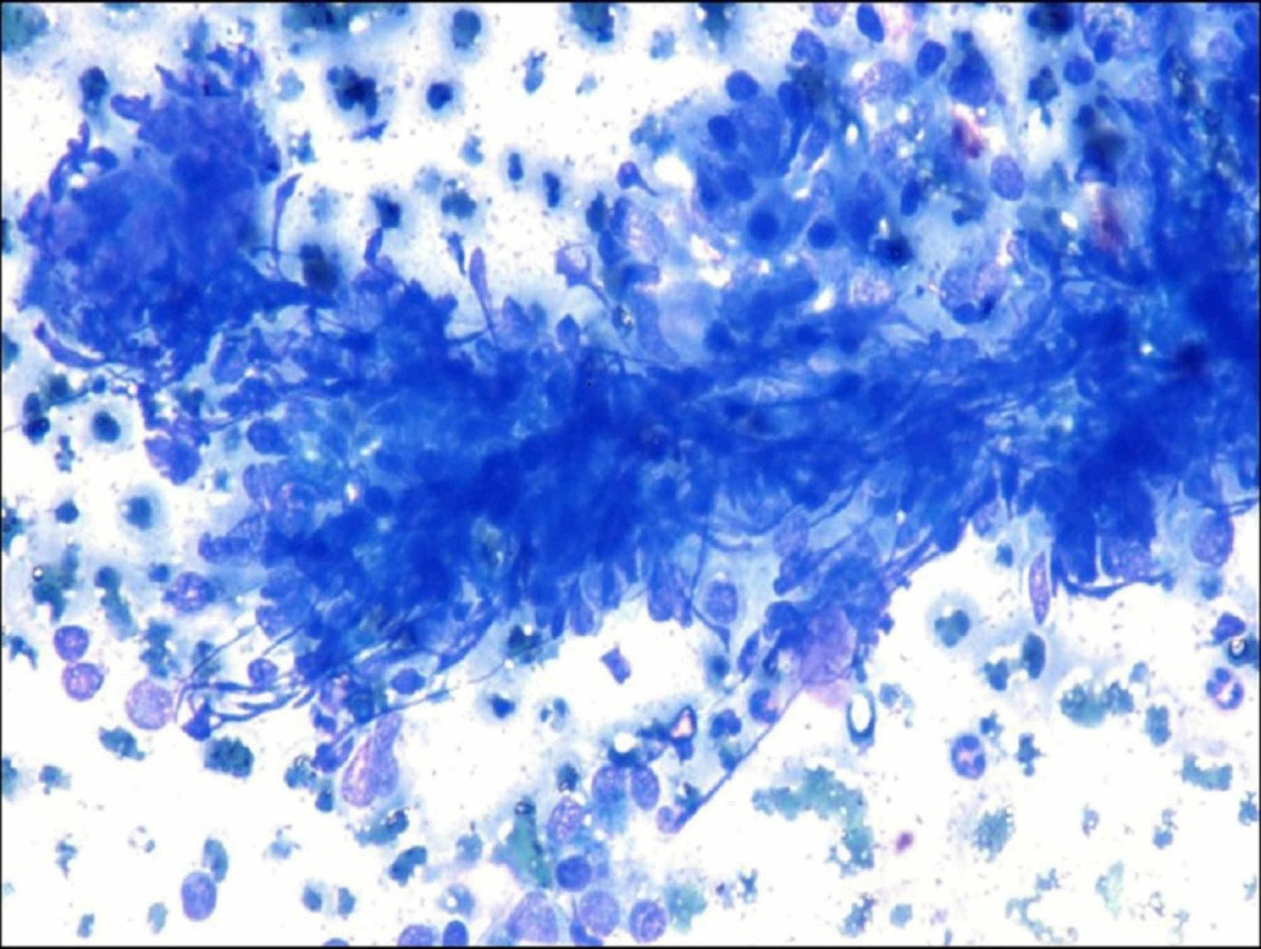
Cytology image Crushed Fragments; (Giemsa stain, x400).

It is a well-known fact that follicular epithelial cells in HT are invaded by lymphocytes. So, the crushed fragments are formed because follicular epithelial cells rendered fragile as a result of the destruction caused by lymphocytes. The crushed fragments are an easily recognizable low power finding. This finding could avoid the false-negative and misdiagnosis of neoplasm in paucicellular and highly cellular smear respectively. So, crushed fragments are a sign, if it is present at low power, one should seek other features of HT. Gandhi et al. [5] used the term crushed artifacts for this feature and they observed that about 82.11% of HT showed the presence of crushed morphology of follicular epithelial cells. In this study, we used the term crushed fragments as it is more appropriate rather than artifacts as this is not something which is occurring due to the methodology of smear preparation. In the present study, 83.33% of HT cases revealed the presence of crushed fragments which is comparable to the results of the study by Gandhi et al. [5] (Figure 5). None in the control group in our study showed this feature. The presence of crushed fragments is statistically significant in this study. So, crushed fragments and eosinophils will be a useful cytological feature in paucicellular and highly cellular smear where a cytological diagnosis is difficult based on established cytological features.

## Conclusions

The presence of eosinophils and crushed fragments as an additional cytological diagnostic feature can improve the efficacy of thyroid FNAC. The crushed fragments, if visible at low power, should make one look for other features of HT. So, the crushed fragments and eosinophils could avoid the false-negative and misdiagnosis of neoplasm in paucicellular and highly cellular smear respectively.
